# Acute Articular Rheumatism Treated with Lemon-Juice

**Published:** 1851-03

**Authors:** 


*Acute Articular Rheumatism treated with Lemon-Juice.*—Dr. Rees, of Guy's Hospital, has employed lemon-juice, in doses of an ounce every fourth hour, in acute articular rheumatism. The administration of this remedy in gout and articular rheumatism suggested itself to Dr. Rees' mind from the fact that the elements of lithic acid may be easily converted into those of urea and carbonic acid by the use of water and oxygen, and he was inclined to trust to the remedy more especially in reference to the necessity for a supply of oxygen. He considered, moreover, that "probably the small proportion of alkaline citrate present in the lemon-juice, which by decomposition during the digestion yields an alkaline carbonate to the blood, might assist in the cure." Dr. Rees advances this *theory* with certain reservations, but he firmly believes that the remedy "assists in obtaining earlier relief than has heretofore been the case in a most distressing malady." "The form of rheumatic disease, in which the greatest benefit seems to have been derived from the lemon-juice, is that of acute rheumatism, and that form of rheumatic affection involving the smaller as well as the larger joints in acute inflammation, and known as rheumatic gout."—*Abridged from Dublin Medical Press,* Nov. 27th, 1850.

